# Role of C5 Activation Products in Sepsis

**DOI:** 10.1100/tsw.2010.216

**Published:** 2010-12-14

**Authors:** Peter A. Ward

**Affiliations:** Department of Pathology, University of Michigan Medical School, Ann Arbor, MI, USA

**Keywords:** C5a, C5a receptors, polymicrobial sepsis, cytokines

## Abstract

Complement activation products are known to be generated in the setting of both experimental and human sepsis. C5 activation products (C5a anaphylatoxin and the membrane attack complex [MAC] C5b-9) are generated during sepsis following infusion of endotoxin, or after cecal ligation and puncture (CLP), which produces polymicrobial sepsis. C5a reacts with its receptors C5aR and C5L2 in a manner that creates the “cytokine storm”, and is associated with development of multiorgan failure (MOF). A number of other complications arising from the interaction of C5a with its receptors include apoptosis of lymphoid cells, loss of innate immune functions of neutrophils (PMNs, polymorphonuclear leukocytes), cardiomyopathy, disseminated intravascular coagulation, and complications associated with MOF. Neutralization of C5a *in vivo* or absence/blockade of C5a receptors greatly reduces the adverse events in the setting of sepsis, markedly attenuates MOF, and greatly improves survival. Regarding the possible role of C5b-9 in sepsis, the literature is conflicting. Some studies suggest that C5b-9 is protective, while other studies suggest the contrary. Clearly, in human sepsis, C5a and its receptors may be logical targets for interception.

## INTRODUCTION

Sepsis in humans is a complex and challenging clinical problem in terms of both diagnosis and treatment. In North America, there are about 600,000 cases per year, with a mortality rate that currently approximates 30%. Since most septic patients are in intensive care units and are often mechanically ventilated, the cost of treating such individuals is extraordinary[1,2]. Improvements in fluid resuscitation and in artificial ventilation have significantly improved outcomes, but reducing mortality rates substantially below the current rates has been a daunting challenge. To date, the only FDA-approved drug is recombinant activated human protein C (APC), which is an anticoagulant, but, like the statins, may have multiple other activities such as anti-inflammatory effects that could explain the ability of APC to improve survival, albeit modestly. The ability of APC to improve survival in sepsis is limited (survival is only improved by about 6%)[3]. In sum, very few, if any, new drugs have been developed that appreciably improve the clinical outcomes in sepsis.

In this report, we will focus on data indicating that robust activation of complement is occurring during sepsis in humans and animals. Because of a growing body of data suggesting that C5a interacting with its receptors (C5aR, C5L2) seems to be linked to many of the adverse outcomes of sepsis, we will briefly review the structures of C5a and its receptors, the consequences of C5a interacting with these receptors to bring about adverse outcomes, and the unresolved question of whether the other major activation product of C5, C5b-9 (MAC, membrane attack complex), plays a significant role in the outcomes of sepsis.

## C5a

Human C5a is a 74-amino-acid glycosylated peptide with antiparallel α-helical structures that are cross-linked by disulfide bands, making the molecule quite stable, especially in the presence of oxidants. C5a is released from the N-terminal region of the α-chain of C5. Such release can occur by the C5 convertases ($C3b_2$●Bb or C4b●C2a●C3b), which cleave C5 into C5a + C5b. C5b interacts with C6, C7, C8, and C9 to form C5b-9 (MAC). It has also been established that C5a can also be released from C5 through the actions of neutral proteases derived from polymorphonuclear leukocytes (PMNs) or lung macrophages[4]. In addition, thrombin (factor IIa) is also effective in causing C5a generation in the presence of C5[5]. C5a is a potent proinflammatory peptide with a variety of biological effects (e.g., increasing vascular permeability and inducing smooth muscle contraction, inducing chemotaxis of PMNs, monocytes, and other cell types) (reviewed [6,7]). In a variety of biological systems, neutralization of C5a or absence of its receptor C5aR dramatically reduces the inflammatory response (as seen in acute lung injury[8] and in organs following ischemia/reperfusion injury[9], and in experimental models of rheumatoid arthritis)[8].

## STRUCTURES AND FUNCTIONS OF C5aR AND C5L2 IN SEPSIS

These receptors are typical seven-membrane-spanning receptors situated on PMNs and macrophages, but also on a variety of other cell types (endothelial cells, alveolar epithelial cells, Kupffer cells, etc.). C5aR is a 45-kDa G protein-coupled receptor (GPCR) that interacts with C5a with very high binding affinity and, to a lesser extent, with C5a des Arg. Signaling of C5aR occurs via MAPKs (p38, ERK1/2, JNK1/2), although the role of each MAPK in various signal transduction responses varies with the cell and with the agonist. There seems to be a consensus that in PMNs, the bulk of MAPK signaling involves RAS, RAF-1, MEK1, and ERK1/2, leading to NF-κB activation, with cytokine/chemokine production and activation of NADPH oxidase (reviewed [10]). PMNs are especially enriched in C5aR, containing around 60,000 high-affinity (e.g., 10 nM) binding sites. After cecal ligation and puncture (CLP) in rats and mice, whole organ extracts (lung, liver, thymus, kidneys, heart) show up-regulation of C5aR, which may be linked to these organs being put at risk of injury or failure during sepsis.

While C5L2 has a molecular weight similar to C5aR, it has three dramatic differences when compared to C5aR: (1) because of a mutation in the DRY region of the third intracellular loop, agonist engagement of C5L2 does not cause an intracellular $Ca^{2+}$ transient[11]; (2) the chief agonists for C5L2 appear to be C5a as well as C5a des Arg and, perhaps, C3a as well as C3a des Arg[12], although the ability of C3a and C3a des Arg to bind to C5L2 and activate cells is a matter of controversy[12,13,14,15,16]; (3) finally, in contrast to C5aR, in the "resting" PMN, the bulk of C5L2 appears to be in the cytosol, present in a granular pattern[15,16]. If the main locale of C5L2 in the nonstimulated PMN is truly in the cytosol, then it is unclear how the agonists for C5L2 can gain cytosolic access to activate intracellular C5L2. Signaling pathways involving C5L2 are also a matter of controversy based on the use of KO mice[13,14,17]. It has been suggested that C5L2 is a "default receptor", competing with C5aR for C5a binding that results in no measureable functional outcome[11]. However, the biological function of C5L2, whether simply to compete with C5aR for C5a binding or whether C5a ligation to C5L2 results in proinflammatory or anti-inflammatory outcomes, is quite unsettled. When human neutrophils were activated with C5a, the expected activation (phosphorylation) of ERK1/2 was found[10]. In the copresence of blocking mAb to human C5L2, this activation process was intensified, suggesting that, ordinarily, C5L2 negatively regulates the activation of ERK1/2[18]. In contrast, the Toronto group reported that LPS or C5a activation of neutrophils resulted in ERK1/2 phosphorylation in

Wt cells, but failed to occur in C5L2$^{-/-}$ cells[13]. In addition, the Gerard group[14] reported that C5L2$^{-/-}$ mice had intensified injury after intrapulmonary deposition of IgG immune complexes, while the Toronto group, using the same model of lung injury, reported greatly reduced intensity of lung injury in C5L2$^{-/-}$ mice[13]. It is not currently possible to reconcile these divergent sets of data. In C5L2$^{-/-}$ mice following CLP, there were substantial increases in serum levels of IL-6 when compared to CLP Wt mice[17], which might be consistent with the idea that, in the absence of C5L2, C5aR can function in an unregulated manner. As related to CLP-induced sepsis in mice, recent data suggest that C5L2 acts collaboratively in conjunction with C5aR to enhance the cytokine storm as well as increased mortality (see below).

## ROLES OF C5a, C5aR, AND C5L2 IN EXPERIMENTAL SEPSIS

### C5a

There is accumulating evidence that in subhuman primate models of sepsis (infusion of live *Escherichia. coli* into monkeys), C5a blockade (by rabbit polyclonal antibody) reduced several metabolic parameters of sepsis and caused diminished evidence of the adult respiratory distress syndrome, although the small group of animals did not permit any statistically significant differences between the groups treated with C5a neutralizing antibody vs. control IgG[19]. In rats or mice with CLP, both rabbit polyclonal neutralizing antibodies and mouse monoclonal antibodies (mAbs) that neutralized C5a were highly effective in attenuating the parameters of sepsis (clinical symptoms, evidence of multiorgan failure [MOF], consumptive coagulopathy, innate immune functions, apoptosis, etc.), resulting in greatly improved survival[20,21]. When administration of mAbs was delayed for as long as 12 h after CLP, there was still substantially improved survival, suggesting that in this experimental model, there is a significant period of time after the onset of sepsis during which intervention to block C5a is effective[22].

It seems clear that excessive C5a generated during sepsis has harmful effects, as described above. Some of the most clear-cut harmful effects of C5a on PMNs in the setting of sepsis are defective chemotaxis, not only to C5a, but to structurally unrelated peptides, such as N-formyl-Met-Leu-Phe (N-fMLP); defective respiratory burst function ($O_2$ consumption and generation of $O_2\cdot$and $H_2O_2$) in PMNs stimulated with phorbol ester; defective phagocytosis; and defective binding of C5a to blood PMNs, but not defective binding of N-fMLP[23,24]. These data suggest that global defects develop in innate immune functions of PMNs in the setting of sepsis. *In vivo* infusion of neutralizing antibodies to rat C5a in CLP rats markedly blocks development of all of these defects in PMNs during sepsis (Fig. 1).

### C5aR and C5L2

Experimental studies in rats or mice with CLP suggest a dynamic balance between C5aR and C5L2 on blood PMNs and in organs. As suggested above, as sepsis progresses, binding sits on PMNs are markedly reduced, as the C5a-C5aR complexes on cell surfaces are internalized into PMNs[25]. The loss of such binding sites correlates with survival: the greater the loss of binding sites, the worse the prognosis for survival. When organs (liver, thymus, lungs, kidneys, heart) were evaluated for mRNA for C5aR and for C5L2, in general there was evidence of up-regulation of mRNAs for these receptors, suggesting that perhaps the organs were being put at risk for injury in the presence of circulating C5a. Immunochemical staining for C5aR in organs during the early phases of sepsis indicated the concomitant up-regulation of protein for C5aR[25,26]. In the case of thymocytes obtained from CLP rats, we found that as early as 3 h after CLP, C5a binding sites on thymocytes were increased. This was associated with preceding up-regulation of C5aR protein on the thymocytes[27]. Such data suggest that in CLP-induced sepsis, binding sites for C5a in organs can be extremely rapidly up-regulated, followed by down-regulation as internalization of C5a●C5a receptor complexes occurs[25].

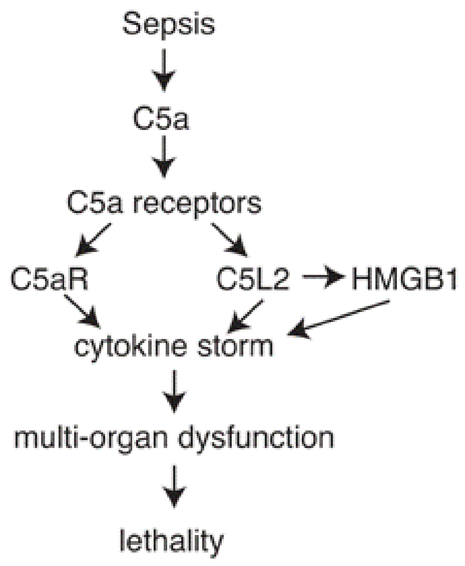

**FIGURE 1.** Sequence of sepsis, complement activation, and lethality. Roles of C5a interacting with its receptors after onset of sepsis induced by CLP in rodents. The downstream effects are the "cytokine storm", together with production of HMGB1 from macrophages, which accentuates the cytokine storm. Ultimately, these events lead to multiorgan dysfunction and lethality.

Our group recently provided a comprehensive report on the role of C5aR and C5L2 in the setting of CLP in mice, using C5a receptor KO mice, neutralizing antibodies to C5a receptors or C5a blockade[20,21,25,27]. Such studies indicate that both C5aR and C5L2 contribute to the cytokine storm, MOF, and lethality. It appeared that >75% suppression of the cytokine storm occurred in the absence of either C5aR or C5L2, suggesting that the appearance of cytokines/chemokines in plasma after CLP occurs via sequential engagement of both receptors[20]. Another finding in this study was that high mobility globulin β1 (HMGB1), which induces release of multiple cytokines and chemokines from macrophages, was produced by LPS-stimulated macrophages via engagement of C5L2, but not C5aR[20]. HMGB1 is known to be important for its role in CLP-induced sepsis[28].

Assuming that C3a des Arg, C5a, and C5a des Arg are ligands for C5L2[11,12,13,29], the issue arises about the role of C3a in the development of CLP-induced sepsis. The most direct data bearing on this question, using $C3^{-/-}$ mice, show that these mice are more susceptible to the lethal effects of CLP[30,31]. Accordingly, it is not clear to what extent C3a/C3a des Arg and C3aR play roles in events following CLP. The reason for increased susceptibility of $C3^{-/-}$ to the lethal effects of sepsis seems likely related to the inability to generate opsonic factors such as C3b and iC3b. $C3^{-/-}$ mice 24 h after CLP had much higher levels of blood CFUs compared to Wt mice[30,31].

## ROLE OF C5b-9 IN CLP-INDUCED SEPSIS

Since C5b-9 is known to have lytic activity for Gram-negative bacteria and because C5b-9 is generated in conditions associated with tissue injury (such as ischemia/reperfusion injury)[9], the role of C5b-9 in CLP-induced sepsis is important to understand since one strategy to block C5a generation during sepsis would be to use a C5-depleting mAb, such as that approved for the treatment of patients with paroxysmal nocturnal hemoglobinemia, which is due to a defect in one of the complement regulatory proteins[32].

The role of C5b-9 in the setting of CLP-induced sepsis is unclear. Depletion of C5 in CLP mice using an antibody to C5 produced protective effects, as did the absence of C6 in CLP rats[33], although to what extent this could be explained by absence of either C5a or C5b-9 remains unclear. Since C6-deficient mice are not available, we recently used a protocol that depletes C6 using a polyclonal antibody to mouse C6[30]. This resulted in ≥80% reduction in CH50 levels in mouse serum. Under such conditions, C6-depleted mice had blood CFU levels 24 h after CLP that were approximately 10 times higher than CFU levels in CLP mice that were C6 intact, suggesting that defective production of C5b-9 might be related to reduced ability to contain bacteremia in these C6-depleted mice. Obviously, it is not clear to what extent C5b-9 is harmful or protective in the setting of CLP-induced sepsis.

## C5a AND APOPTOSIS IN SEPSIS

It has been well documented that sepsis in humans and rodents induces a state of immunosuppression that is related to an early loss of both B and T cells in lymphoid tissues and in blood[27,34,35]. Lymphoid cell loss appears to be due to induction of apoptosis of these cells, likely via engagement of both the intrinsic (mitochondrial) and extrinsic (TNFα, Fas ligand) pathways of apoptosis. Our own studies have focused on apoptosis of thymocytes in CLP rats. Sepsis induced a striking loss of mass of the thymus (>50%) in the first few days after CLP. C5aR content on thymocytes was found to rise rapidly after CLP and increased binding of C5a to thymocytes could be found as early as 3 h after CLP. Furthermore, *in vitro* exposure of C5a to CLP thymocytes induced apoptosis *in vitro*, featuring activation of caspases-3, -6, and -9. The use of neutralizing antibody to C5a given at the time of CLP also preserved thymic mass and largely abolished evidence of caspase activation in and apoptosis of thymocytes[27,35]. These studies suggest that CLP induces a rapid and early response (up-regulated) of C5aR on lymphoid cells.

When sepsis in both humans and animals becomes progressive, septic shock usually ensues. We reasoned that such outcomes might be related to inadequate reserves of epinephrine and norepinephrine released from the adrenal medulla. In a recent study of CLP in mice, we found extensive apoptosis of adrenal medullary cells 24 h after CLP. When dual blockade of both C5aR and C5L2 was accomplished using antibodies, the degree of apoptosis of adrenal medullary cells was greatly diminished, as assessed by the TUNEL assay[36]. Using the PC12 cell line derived from a human pheochromocytoma, addition of C5a totally suppressed the release of norepinephrine and dopamine[36]. This was shown to be coincidental with the fact that C5a caused apoptosis of the PC12 cells. Such data suggest that C5a released after CLP-induced sepsis may lead to adrenal medullary cell apoptosis, predisposing animals to the development of septic shock.

## C5a EFFECTS ON THE HEART DURING SEPSIS

Development of reversible cardiac dysfunction (biventricular dilation, increased cardiac output followed by falling output, decreased systemic vascular resistance, etc.) commonly occurs as sepsis advances. "Septic cardiomyopathy" has been defined by *in vitro* dysfunction of cardiomyocytes (CMs). When CMs were isolated from septic rodents, they exhibited defective contractility as well as defective relaxation[37]. Such functional defects would imply that the heart has lost its resilience and ability to adjust during sepsis in order to maintain sufficient cardiac output and maintain adequate blood pressure for organ perfusion. Furthermore, when CMs from normal rats were incubated *in vitro* with C5a, these cells developed dramatic defects in spontaneous contractility and relaxation, associated with the fact that normal CMs contain constitutive C5aR on their cell surfaces[37]. When C5a was blocked *in vivo* (with neutralizing antibody) at the time of CLP, CMs obtained from these animals after CLP showed full contractility and relaxation functions, indicating that C5a produced during CLP may directly cause defects in cardiac function. "Cardiosuppressive cytokines" (IL-1β, TNFα, IL-6, etc.) have been described in plasma from septic humans[38,39]. The definition of such cytokines was based on their ability to cause contractile defects in normal CMs. Whether this finding can be linked to the effects of C5a on CMs remains to be seen.

## CONCLUSIONS

It has been known for some time that septic humans demonstrate C5a in their plasma (10–100 nM), that there are corresponding falls in serum complement hemolytic activity (CH50), and that under these conditions, defective innate immune functions occur in blood PMNs[23]. Similar defects can be mimicked by *in vitro* exposure of normal blood PMNs to C5a[22,24]. In an accumulating body of evidence, polymicrobial sepsis induced in rodents by CLP results in a series of cell and organ dysfunctions that can be linked to C5a and its receptors C5aR and C5L2. Use of blocking antibodies to C5a or to C5a receptors, or use of synthetic compounds that block C5a receptors, dramatically attenuate most of the complications of sepsis (Figs. 1 and 2). These data suggest that in humans with sepsis, consideration should be given to a strategy designed to either block C5a or its receptors.

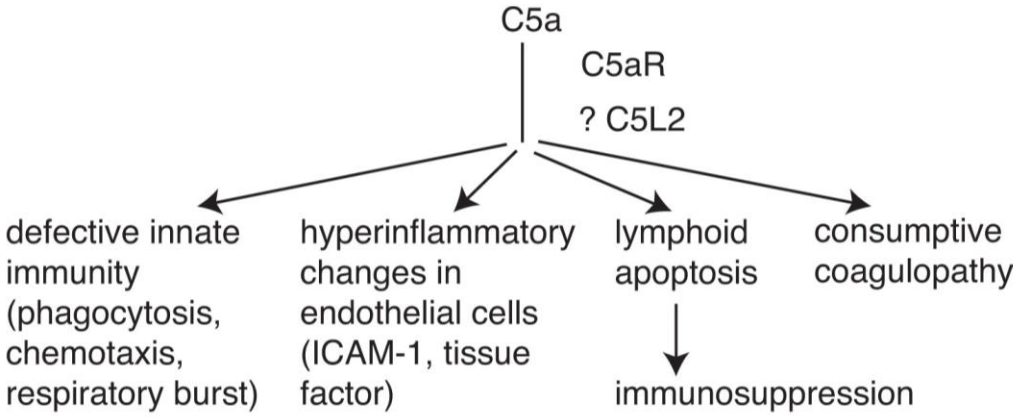

**FIGURE 2.** C5a generated during sepsis interacts with its receptors C5aR and C5L2. This leads to four major complications: defects in innate immune responses of neutrophils, conversion of endothelial cells to a hyperinflammatory state with increased expression of ICAM-1 and tissue factor, lymphoid cell apoptosis with ensuing immunosuppression, and consumptive coagulopathy. All of these complications can be blocked by *in vitro* blockade of C5a or its two receptors.

## ACKNOWLEDGMENTS

This work was funded by NIH grant GM-61656. The author has no potential or competing financial interests.
